# Editorial: Adrenal Cortex: From Physiology to Disease

**DOI:** 10.3389/fendo.2016.00051

**Published:** 2016-06-01

**Authors:** Pierre Val, Antoine Martinez

**Affiliations:** ^1^UMR6293 GReD, Molecular Pathophysiology of Adrenal and Endocrine Tissues, CNRS, Aubiere, France

**Keywords:** adrenal, development, physiology, zonation, disease, benign tumour, cancer, insufficiency

**The Editorial on the Research Topic**

**Adrenal cortex: from physiology to disease**

The adrenal gland plays essential roles in the control of body homeostasis, stress, and immune responses. The adrenal cortex represents up to 90% of the gland and is specialized in the production of adrenal steroids. The coordinate production of these steroids relies on adrenal cortex zonation, which corresponds to the establishment of distinct concentric functional zones in the perinatal period: outermost zona glomerulosa synthesizes mineralocorticoids, zona fasciculata produces glucocorticoids, and innermost zona reticularis synthesizes both glucocorticoids and adrenal androgens.

This zonal organization has to be maintained throughout the life of the individual, despite constant centripetal tissue renewal. The review by Pihlajoki et al. summarizes the latest findings on the mechanisms of adrenal cortex renewal, which relies on outer cortex progenitors recruitment and lineage conversion along cell migration within the cortex. This paper also provides a comprehensive overview of the hormones, signaling pathways, and transcription factors that control these processes to allow for on-demand adaptation of cortical function and maintenance of adrenal homeostasis.

Defects in adrenal development and maintenance are associated with adrenal insufficiency, a life threatening condition for which lifelong hormonal replacement therapies can be challenging. The review by Ruiz-Babot and colleagues sheds light on novel developments in the field of adrenal replacement, including pluripotent cell reprograming and the use of encapsulating devices with semi-permeable membranes to avoid immune rejection of grafts. These promising approaches could pave the way for future clinical management of adrenal-insufficiency patients.

While adrenal insufficiency is clinically problematic, the opposite situation in which adrenal steroid production is increased also raises significant clinical concerns. Hypercortisolism results in Cushing’s syndrome associated with central obesity, arterial hypertension, immunosuppression, and depression. Hyperaldosteronism is associated with high blood pressure and profound cardiovascular and renal alterations, which result in increased risk of cardiovascular failure. These highly morbid syndromes are the consequence of either benign hyperplasia and tumors or adrenocortical cancer (ACC).

The review by Boulkroun and colleagues establishes the molecular bases of normal control of aldosterone production and elaborates on recent next-generation sequencing (NGS) analyses that allowed identification of mutations in potassium and calcium channels as key players in the development of hyperaldosteronism. Even if these mutations can explain increased aldosterone secretion, they are unlikely to account for tumor growth. Boulkroun et al. summarize data showing that deregulated cell growth in aldosterone-producing adenomas is likely to result from WNT and SHH signaling pathway activation in these tumors.

Deregulated protein kinase A (PKA) signaling is a common theme in adrenal tumors associated with ACTH-independent hypercortisolism. These include primary pigmented adrenocortical disease (PPNAD), adrenal adenomas, bilateral macronodular adrenal hyperplasia (BMAH), and adrenal cancer. The review by Berthon et al. provides in-depth insight into the genetic causes of deregulated PKA signaling, including inactivating *PRKAR1A* mutations in PPNAD and activating *PRKACA* mutations in cortisol-producing adenomas. Interestingly, mutations in either *PRKAR1A* or *PRKACA* were not found in BMAH. The review by Drougat et al. emphasizes the discovery of mutations in *ARMC5* as a likely cause of these particular benign adrenal tumors and elaborates on potential pathogenic mechanisms. Lefebvre et al. shed another light on BMAH by focusing on the paracrine regulation of cortisol secretion. They gather data showing that cortisol secretion is stimulated by the release of a number of factors either produced by non-steroidogenic cells within the cortex (mast, chromaffin, and endothelial cells) or by a subset of aberrantly differentiated steroidogenic cells that can release serotonin or even ACTH within the hyperplastic tissue. They further suggest that aberrant ACTH production and expression of ectopic receptors, such as the receptors of LH, GIP, and 5-HT7, may be the result of aberrant differentiation of gonadal-like cells, triggered by driver mutations, such as ARMC5 inactivation.

Beyond steroid hormone excess, which is also observed in about 40–60% of patients, ACC still represents a major therapeutic challenge. The review by Libé and colleagues provides an overview of current diagnosis and treatment of ACC, which emphasizes the need for novel therapeutic targets in a cancer with dismal prognosis. The review by Drougat and colleagues provides insight into the role of mutations targeting the WNT signaling pathway (essentially activating mutations of *CTNNB1* and deletions of *ZNRF3*) and their pathogenic role in ACC. This paper on adult ACC is nicely complemented by Lalli and Figueiredo’s review that focuses on pediatric ACC. These are rare tumors that generally occur in the context of *TP53* alterations, in particular the specific R337H mutation found with high frequency in Southern Brazil. The authors present evidence that these tumors are likely to derive from the fetal adrenal and discuss the common and divergent alterations found in pediatric and adult ACC, which highlights the lack of effective prognosis markers in the former. Deregulation of miRNA production is a common theme in most cancers. Nadia Cherradi provides a comprehensive overview of miRNA deregulation in ACC and shows that they can provide novel insight into the pathogenesis of ACC and may constitute interesting therapeutic targets. This review also highlights the usefulness of circulating miRNAs as novel non-invasive diagnostic and prognostic biomarkers in ACC. It further elaborates on an exciting aspect of miRNAs biology that involves their circulation within ACC cell-derived exosomes, which would allow communication with tumor microenvironment.

We hope that you will find this topic inspiring and that it will shed light on exciting aspects of adrenal physiology and disease.

## Author Contributions

All authors listed, have made substantial, direct and intellectual contribution to the work, and approved it for publication.

## Conflict of Interest Statement

The authors declare that the research was conducted in the absence of any commercial or financial relationships that could be construed as a potential conflict of interest.

